# High risk HPV genotypes in Belarus

**DOI:** 10.1186/1471-2334-14-S2-P98

**Published:** 2014-05-23

**Authors:** VF Eremin, GI Viarheichyk, EL Gasich, EA Shishkin, MV Domnich, EI Nesterovskaja

**Affiliations:** 1 Republican research and practical center of epidemiology and microbiology, Minsk, Belarus; 2 Gomel state medical University, Gomel, Belarus

## Introduction

897 cases of cervical cancer and 358 women death are registered in Belarus for 2008.

## Materials and methods

764 PCR positive vaginal and cervical specimens. PCR,products were purified and sequenced on the genetic analyzer ABI Prism 3100 Avant. Software programs Sequencing Analysis 5.1.1., SeqScape v2.6, BioEdit were used. Phylogenetic analysis was performed using the MEGA4.1 software with the Neighbour-Joining and Kimura 2-parameter method.

## Results

Of 764 PCR positive samples there were sequenced 77 and 10 DNAs on genes L1 (296 bp) and E6/E7 (620 bp) HPV-16 regions, respectively. The phylogenetic analysis of gene L1 region established that generally all samples from Belarus clustered round reference-sequences from South East Asia and Europe, only 15 and 9 samples formed independent groups with each other. p-distances between the Belarusian samples fluctuated from 0.000 to 0.024 that points as to new virus penetration to the country, and on circulation of our "old" HPV-16 types which formed independent groups of samples on a phylogenetic tree. Separately the sample 63 from Gomel settled down, it didn't enter one of groups. Most likely, it is our "domestic" HPV-16 variant. Three viruses, two from Minsk and one from the Grodno region settled down in one group "Africa" with African reference - sequences, but separately from each other that points to a different origin of a virus at these patients. Sequencing on gene of E6 and E6/E7 region also divided viruses into groups. Also as well as on L1 gene on gene E6/E7 region virus formed, generally the general groups with Asian HPV-16 variants. In too time part of isolates settled down separately and formed independent groups.

## Conclusion

Thus, carried-out sequencing and phylogenetic analysis have showed that on the territory of Belarus circulation as "old" HPV-16 variants takes place, and there is a penetration of new types HPV because of borders of Belarus, generally from the countries of South East Asia.

